# Seroprevalence of syphilis and human immunodeficiency virus infections among pregnant women who attend the University of Gondar teaching hospital, Northwest Ethiopia: a cross sectional study

**DOI:** 10.1186/s12879-015-0848-5

**Published:** 2015-03-03

**Authors:** Mengistu Endris, Tekalign Deressa, Yeshambel Belyhun, Feleke Moges

**Affiliations:** School of Biomedical and Laboratory Sciences, College of Medicine and Health Sciences, University of Gondar, P.O.Box.196, Gondar, Ethiopia; Institute of Virology Faculty of Medicine, University of Leipzig, Johannisallee 30, 04103 Leipzig, Germany

**Keywords:** HIV, Syphilis, Pregnant women, Antenatal care

## Abstract

**Background:**

Syphilis and HIV infections in pregnancy result in a number of adverse outcomes including neonatal death and vertical transmission. Ethiopia is a country where these infections are highly prevalent. However, data on co-morbidities of syphilis and HIV among pregnant women in Gondar are scarce. Thus, the aim of this study was to determine the seroprevalence of these infections and associated factors among pregnant women attending antenatal care at the University of Gondar teaching hospital, Northwest Ethiopia.

**Methods:**

A cross sectional study was conducted from February to June 2011. Structured interviews were used to collect socio-demographic and obstetric data. Sera against syphilis were screened by rapid plasma reagin test; and confirmed by *Treponema pallidum* hemagglutination assay. HIV infection was detected by rapid HIV test kits following the national algorithms for HIV testing. Data were summarized by descriptive statistics and binary logistic regression. Odds ratio (OR) and 95% confidence intervals (CI) were calculated.

**Results:**

Of 385 pregnant women, reactive syphilis was noted in 11/385 (2.9%) and seroprevalence of HIV was 43/385 (11.2%). The prevalence of syphilis and HIV co-infection was 2/385(0.5%). High rate of syphilis was observed among the women with above 30 years of age (OR 3.69, 95% CI 0.83 - 16.82). Women with a history of miscarriage and stillbirth were more likely to be infected by syphilis (OR 2.22, 95% CI 0.54-9.60) and (OR 3.24, 95% CI 0.00-17.54), respectively.

**Conclusion:**

Our data indicated that syphilis and HIV infections are still important public health concerns among pregnant women in the Gondar area. Hence, we recommend strenuous screening of all pregnant women for these infections during antenatal care. Further, strengthening health education on the mode of transmission and prevention of HIV and syphilis is essential for effective control of these infections.

## Background

Syphilis and HIV infections continue to be a public health problem in the world, especially in developing countries. Both infections are transmitted by unprotected sex, unsafe blood transfusion, needle sharing and from mother to child *in utero* [1,2]*.* In pregnancy, these infections have been associated with a number of adverse outcomes including miscarriage, stillbirth, neonatal death, low birth weight and congenital infections [3-6]. In addition, ulcerative genital lesions caused by syphilis have been associated with increased HIV acquisition and transmission. HIV, on the other hand, influences the clinical presentations and treatment outcome of syphilis by favoring escape of the *Treponema* from the host immune response [7-9].

The World Health Organization (WHO) estimates that over 1.4 million pregnant women are being affected by syphilis and HIV in the world every year [1,4-6]. Untreated syphilis and HIV in pregnancy have been reported to cause adverse pregnancy outcomes in about 50% and 20% cases respectively [10]. Early diagnosis and treatment of pregnant women who tested positive for syphilis have been shown to be effective in reducing stillbirth, neonatal death and congenital infection by more than 55% [11,12]. Importantly, these interventions have been estimated to be highly cost-effective even in low income countries [11].

Ethiopia is one of the countries where syphilis and HIV infections are highly prevalent. The estimated adult prevalence of HIV had been 2.4% in 2009 [13,14]. According to this report, there are about 90,000 HIV-positive pregnant women with an estimated 14,000 HIV-positive births and 28,000 AIDS orphans. The prevalence of syphilis among antinatal care (ANC) attendants had been 2.3% in 2009 [15]. The prevalence of syphilis and HIV infections has been shown to vary by geographic areas, study population and time-period [16-19]. Thus, obtaining periodical estimates of the local epidemiological picture of these infections by monitoring various risk groups is essential for guiding clinical action, resource allocation, and intervention protocols to make progress toward elimination.

To this end, the current study reports the seroprevalence of syphilis and HIV infections, and associated risk factors among pregnant women attending a routine ANC clinic at the University of Gondar teaching hospital, Northwest Ethiopia.

## Methods

### Study design, study site and population

This cross-sectional study was conducted at the University of Gondar teaching hospital, Gondar, Northwest Ethiopia. The hospital is a tertiary level teaching hospital that provides medical service to about five million people in Northwest Ethiopia. Gondar is located in North of Lake Tana and Southwest of the Siemen Mountains. It has a latitude and longitude of 12°36’N 37°28’E with an elevation of 2133 meters above sea level. The study population was all pregnant women who attended the ANC clinic of the University of Gondar teaching hospital from February to June 2011.

### Data collection tool

Information regarding socio-demographic and obstetric characteristics of the study participants was collected using pre-tested questionnaires. In the questionnaire, data like marital status, residence, self-reported history of prior still birth and/or miscarriage, occupation and education levels were included.

### Laboratory methods

Five ml of venous blood was obtained in vacutainer tubes from each study participants as part of a routine ANC and used for sera preparation. The sera were stored at 4°C. The sera were screened for HIV using a KHB diagnostic kit. Confirmatory tests of reactive samples were performed using Stat-Pack and Uni-Gold kits as per the national algorism for HIV testing. Syphilis seropositivity was tested by using rapid plasma reagin (RPR) (Omega Immutrep-RPR®, UK) following the manufacturer’s instructions. Reactive samples to RPR were confirmed by using *Treponema pallidum* hemagglutination assay (TPHA) (Omega Immutrep TPHA®, UK). Samples tested positive by both diagnostic kits were considered as active or untreated syphilis.

### Statistical analysis

Data were entered, cleaned and analysed using SPSS version 20 statistical package (SPSS, Chicago, IL, USA). Cleaning of data was done to check the consistency and completeness of the data set. Frequencies and proportions were used to describe the study population in relation to relevant variables. Bivariate logistic regression was used to identify significant predictors. The degree of association between independent and dependent variables was assessed using odds ratio with 95% confidence interval. P value of 0.05 was considered as statistically significant.

### Ethical statements

Ethical clearance was obtained from the Institutional Ethical Review Board of the University of Gondar. After explaining the objectives of the study, written consent was obtained from each study participant. For the participants who were unable to read and write, trained interviewers fully explained the purpose, benefits, and potential risks before consent was obtained. In this case, finger print was used as a signature. The interviews with study participants were conducted with strict privacy and assuring confidentiality.

## Results

A total of 385 pregnant women were enrolled to the study. All women approached for participation were consented, interviewed and provided clinical samples (participation rate = 100%).

### Demographics

The median age of the study population was 25 years (SD ± 5.4). Two hundred thirty seven women (61.6%) were in the age group of 21–29 years. The large proportion of the women 378 (98.2%) were married and 254 (66%) of them were multigravida. About one-third (33.2%) of the women were illiterate. Of all the study participants, 276 (71.7%) were living in urban settings and 350 (90.9%) were orthodox Christians. With respect to occupation, 71.2% of the women were house wives, followed by employed (20.5%), merchants (4.9%) and students (3.4%). Of the 385 women, only 20 (5.2%) were screened for syphilis and HIV in their first trimester; whereas 254 (66%) were screened in their second trimester (Table 1).

**Table 1:** **Socio-demographic and obstetric characteristics of pregnant women attending ANC clinic at the university of Gondar teaching hospital from February to June 2011**

**Characteristics**	**Number (n = 385)**	**Percent (%)**
Age category		
<20	64	16.6
21-29	237	61.6
≥30	84	21.8
Residence		
Urban	276	71. 7
Rural	109	28.3
Marital Status		
Married	378	98. 2
Single/divorced/widowed	7	1. 8
Gravidity		
primagravida	131	34. 0
Multigravida	254	66. 0
Gestational stage at screening		
First trimester	20	5.2
Second trimester	254	66.0
Third trimester	111	28.8
Prior stillbirth	26	6.8
Prior miscarriage	57	14.8
Religion		
Orthodox	350	90. 9
Muslim	35	9. 1
Occupation		
Employed	79	20. 5
Housewife	274	71. 2
Student	13	3.4
Merchant	19	4.9
Education		
Illitrate	128	33.2
Elementary	89	23.1
Secondary and above	168	43.6

### Seroprevalence of syphilis and associated risk factors

The overall prevalence of syphilis was 11/385(2.9%). All syphilis seropositive cases were observed among married women who were house wives by occupation (Table 2). In terms of age, high syphilis seroprevalence was observed among women in the age group of above 30 years (6%). Among syphilis seropositive women, a higher proportion (54.5%) were screened for the first time at third trimester of their pregnancy.

**Table 2:** **Bivariate analysis of associated factors for Syphilis seropositivity among pregnant women from February to June 2011**

**Characteristics**	**Syphilis**		**COR (95%CI)**	**P-value**
	**Positive**	**Negatives**		
Age category				
<20	2(3.1)	62(96.9)	1.88 (0.23-12.29)	0.37
21-29	4(1.7)	233(98.3)	1.00	---
≥30	5(6.0)	79(94)	3.69 (0.83-16.82)	0.06
Residence				
Urban	6(2.2)	270(97.8)	1.00	---
Rular	5(4.6)	104(95.4)	2.16 (0.56-8.22)	0.17
Marital Status				
Married	11(2.9)	367(97.1)	Undefined	0.81
Single/divorced/widowed	0(0.0)	7(100)	1.00	---
Gravidity				
Primagravida	1(0.8)	130(99.2)	1.00	---
Multigravida	10(3.9)	244(96.1)	5.33 (0.69-112.46)	0.07
Gestational stage at screening				
First trimester	0(0.0)	20(5.3%)	1.00	---
Second trimester	5(2.0%)	249(98%)	Undefined	0.68
Third trimester	6(5,4%)	105(94.6%)	Undefined	0.36
Stillbirth				
Yes	2(7.7)	24(92.3)	3.24(0.00-17.54)	0.17
No	9 (2.5)	350(97.5)	1.00	---
Miscarriage				
Yes	3(5.3)	54 (94.7)	2.22 (0.54-9.60)	0.21
No	8(2.4)	320(97.6)	1.00	---
Occupation				
Employed	0(0)	79(100)	1.00	---
Housewife	11(4.0)	263(96.0)	Undefined	0.05
Student	0(0.0)	13 (100)	Undefined	NA
Merchant	0(0.0)	19(100)	Undefined	NA
Education				
Illitrate	4(3.1)	124(96.9)	0.23 (0.02-6.03)	0.26
Elementary	6(6.7)	83(93.3)	0.51 (0.05-12.73)	0.46
Secondary and above	1(0.6)	167(99.4)	1.00	---
**Over all**	**11(2.9)**	**374 (97.1)**		

COR: crude odds ratio, NA: Not applicable.

Bivariate analysis revealed that older women above the age of 30 years (COR 3.69, 95% CI 0.83 – 16.82, P = 0.06), house wives (P = 0.05), multigravida (COR 5.33, 95% CI 0.69 – 112.46, P = 0.07) and rural dwellers (COR 2.16, 95% CI 0.56 - 8.22, P = 0.17) were more likely to be positive for syphilis infection. An experience of miscarriage (95% CI 0.54-9.60, P = 0.21) and a history of stillbirth (95% CI 0.00-17.54, P = 0.17) were associated with syphilis infection with the odds of 2.22 and 3.24 respectively.

### Seroprevalence of HIV and associated factors

Overall, 11.2% (43/385) of the pregnant women were positive for HIV. The prevalence rate of HIV for all socio-demographic groups was ranging from 7.7% to 28.6% (Table 3). In this study, married women were 3.29 times more likely to be infected with HIV (95% CI 0.43 -20.00) than unmarried women. Being merchants was associated with 2.07 times greater odds to HIV infection (95% CI 0.46 – 8.85).

**Table 3:** **Bivariate analysis of associated factors for HIV seropositivity among pregnant women from February to June 2011**

**Characterstics**	**HIV**		**COR (95%CI)**	**P-value**
	**Positive**	**Negative**		
Age category				
<20	7(10.9)	57(89.1)	0.96 (0.36-2.45)	0.90
21-29	27(11.4)	210(88.1)	1.00	---
≥30	9(10.7)	75(89.3)	0.93 (0.39-2.19)	0.97
Residence				
Urban	32(11,6)	244(88,4)	1.00	---
Rular	11(10.1)	98(89.9)	0.86 (0.39-1.85)	0.67
Marital Status				
Married	41(10.8)	337(89.2)	3.29 (0.43-20.00)	0.18
Single/divorced/widowed	2(28.6)	5(71.4)	1.00	---
Gravidity				
Primagravida	12(9.2)	119(90.8)	1.00	---
Multigravida	31(12.2)	223(87.8)	1.38 (0.65-2.96)	0.47
Gestational stage at screening				
First trimester	3(15%)	17(85.0%)	1.00	---
Second trimester	33(13%)	221(87%)	0.85 (0.22-3.85)	0.50
Third trimester	7(6.3%)	104(93.7%)	0.38 (0.08-2.08)	0.18
Stillbirth				
Yes	5 (19.2)	21 (80.8)	2.01 (0.62-6.08)	0.15
No	38(10.6)	321(89.4)	1.00	---
Miscarriage				
Yes	11(19.3)	46 (80.7)	2.21 (0.97-4.96)	0.03
No	32 (9.8)	296(90.2)	1.00	---
Occupation				
Employed	9(11.4)	70(88.6)	1.00	---
Housewife	29(10.6)	245(89.4)	0.92 (0.39-2.20)	0.83
Student	1(7.7)	12(92.3)	0.65 (0.03-5.93)	0.57
Merchant	4(21.1)	15(78.9)	2.07 (0.46-8.85)	0.22
Education				
Illitrate	12(9.4)	116(90.6)	0.81 (0.35-1.84)	0.72
Elementary	12(13.5)	77(86.5)	1.22 (0.53-2.81)	0.75
Secondary and above	19(11.3)	149(88.7)	1.00	---
**Overall**	**43(11.2)**	**342(88.8)**		

COR: crude odds ratio.

HIV and syphilis co-infection was observed in two house wives (0.5%) with the age of 27 and 28 years (data not shown). But, the association between HIV and syphilis seropositivity was not statistically significant (P > 0.05).

## Discussion

Syphilis remains a major cause of morbidity and mortality in the world despite the availability of effective treatment. Interventions including early ANC, massive screening and prompt treatment with antibiotics have been reported to reduce syphilis attributable-risks [11,12]. Ethiopia is among the countries where syphilis and HIV are highly prevalent. In the past decade, ANC coverage in Ethiopia and screening of pregnant women for syphilis as well as HIV has increased markedly [14,15]. Although, information regarding syphilis and HIV prevalence among this group of population from different parts of the country is available, data on co-morbidities of these infections among pregnant women in the Gondar area is scarce. This study sought to address this gap by updating the prevalence of syphilis and HIV among pregnant women attending the ANC at the University of Gondar teaching hospital.

The overall seroprevalence of syphilis among pregnant women was 2.9% which is consistent with the report from Addis Ababa [20]. Compared to other countries, the observed 2.9% seroprevalence of syphilis is higher than the 0.07% prevalence rates in Nigeria [21], 1.6% in Tanzania [22] and 0.39% in rural China [23]. Yet, it is lower when compared with other cities in Ethiopia; the 13.7% seroprevalence in Debretabor [24], 12.1% in Jimma [25] and 4.6% in Gambella [15]. The lower prevalence of syphilis among the study population could be attributed to the increase in syphilis screening at the ANC and effective treatment with antibiotics in the last 10 years [15].

In this study, we found that older age and being from rural areas were associated with syphilis infection. This was in agreement with the national ANC sentinel report that revealed high syphilis infection among women between the age of 34–49 years and rural dwellers [15]. This result was also consistent with the studies from China [23], Zimbabwe [26] and Mexico [27] that displayed higher syphilis prevalence among older population. All syphilis cases were observed among house wives with no/low education. However, it is not clear whether these finding was due to risky sexual behavior of the women or due to the high proportion of house wives in the study. It appears that high rate of syphilis in this group might be due to a risk of re-infection from their partners, as using protective methods like condom is uncommon among married couples. Further**,** it might be partially explained by the fact that these women were less educated and presumably had low treatment seeking behavior [28,29]. However, similar observations were not made with respect to HIV infection that shares a common route of infection with syphilis. Thus, we recommend a further investigation with larger population size to address this issue.

Our data showed that syphilis seropositive women had higher likelihood of experiencing adverse pregnancy outcomes, such as stillbirth and miscarriage. This result is in agreement with a number of previous studies that reported adverse pregnancy outcomes in over half of pregnant women with untreated syphilis [4-6]. Importantly, 54.5% of syphilis seropositive women were screened at the third trimester suggesting that there is a potential risk to experience adverse outcomes. Taken together, this observation highlights the need to strengthen the existing ANC, and timing the interventions to reduce syphilis related adverse pregnancy outcomes.

Our study found high prevalence of HIV infection (11.2%) among pregnant women attending the ANC. This rate was exceeding the 9.6% rate reported seven years ago from this area [16] and the national prevalence of HIV among similar population [14]. But, it is similar to the 11.9% report by Tiruneh [30] and the 11.8% prevalence of HIV among street dwelling women in Gondar [31]. This data indicating that the HIV epidemic remains an important public health concern among pregnant women in the Gondar area and suggest that there is a need to strengthen intervention efforts including voluntary counselling and testing, and health education on prevention of HIV infection.

Analysis of socio-demographic factors revealed relatively higher association of married women and merchants with HIV infection, albeit the correlation did not reach statistical significance. In contrast to a number of studies that have revealed the correlation between syphilis seropositivity and HIV infection [7-9], we have not observed a significant association between the two infections in this study. This could be attributed to the low prevalence of syphilis among the study population.

This study has some limitations in that it includes only those pregnant women who attend the ANC. Thus, the reported figure may underestimate the prevalence of syphilis as a significant number of pregnant women who have no access to ANC and may not seek treatment are missing. Further, we cannot confirm whether the self-reported history of adverse pregnancy outcomes among syphilis seropositive women were due to syphilis infection. Despite the limitations, this study has provided useful data on seroprevalence of syphilis and HIV among pregnant women who attend ANC at the University of Gondar teaching hospital.

## Conclusions

There is still the problem of syphilis and HIV in our region as shown by our findings. The gap and the need we found is that it is important to screen all pregnant women for these infections during the ANC. Thus, it is essential to strengthen the existing ANC services and health education on transmission and prevention of these diseases.
